# Microbial fertilizer regulates C:N:P stoichiometry and alleviates phosphorus limitation in flue-cured tobacco planting soil

**DOI:** 10.1038/s41598-023-37438-w

**Published:** 2023-06-24

**Authors:** Junna Feng, Lulu Chen, Tiyuan Xia, Yanan Ruan, Xiaolu Sun, Tian Wu, Yu Zhong, Xiaodong Shao, Zuoxin Tang

**Affiliations:** 1 College of Agricultural and Life Sciences, Kunming University, Kunming, 650214 Yunnan China; 2 Key Laboratory of Ecosystem Network Observation and Modeling, Center for Forest Ecosystem Studies and Qianyanzhou Ecological Station, Institute of Geographic Sciences and Natural Resources Research, Chinese Academy of Sciences, Beijing, 100101 China; 3 College of Plant Protection, Yunnan Agricultural University, Kunming, 650201 Yunnan China; 4 Agronomy College, Qingdao Agricultural University, Qingdao, 266000 Shandong China; 5 Honghe Branch of Yunnan Tobacco Company, Mile, 652300 Yunnan China

**Keywords:** Biogeochemistry, Ecology, Environmental social sciences

## Abstract

Fertilization can be optimized and managed during the flue-cured tobacco growing period by studying the response of soil and microbial biomass stoichiometric characteristics to fertilization. In this study, we investigated the effect of compound fertilizers combined with microbial fertilizer treatments on the stoichiometric characteristics of the rhizosphere soil and the limitations of microbial resources during the flue-cured tobacco growing period. The results indicated that soil and microbial C:N:P varied greatly with the growing period. The effect of sampling time was usually greater than that of fertilization treatment, and microbial C:N:P did not vary with the soil resource stoichiometric ratio. The microbial metabolism of the tobacco-growing soil was limited by phosphorus after extending the growing period, and phosphorus limitation gradually increased from the root extension to the maturation periods but decreased at harvest. The rhizosphere soil microbial nitrogen and phosphorus limitations were mainly affected by soil water content, soil pH, microbial biomass carbon, and the ratio of microbial biomass carbon to microbial biomass phosphorus. Applying microbial fertilizer reduced phosphorus limitation. Therefore, applying microbial fertilizer regulated the limitation of microbial resources by affecting the soil and microbial biomass C:N:P in flue-cured tobacco rhizosphere soils.

## Introduction

Carbon (C), nitrogen (N), and phosphorus (P) play pivotal roles in regulating plant growth and soil nutrient cycling, and their interactions are closely intertwined through a series of physical, chemical, and biological processes^1,2^. Ecological stoichiometry is an effective and integrated way to study this coupling and any changes in element ratios^3^. The ecological stoichiometric ratio of C, N, and P reflects the relationship between the soil, microorganisms, and enzymes, and has been widely used to study the nutrient supply and demand balance in different ecosystems^4–7^.

Soil C:N:P stoichiometry is a functional trait that reflects nutrient use efficiency and nutrient limitations^8^, and maintains ecosystem functions in response to global change^9^. In plant-soil systems, the soil C:N:P ratio regulates the microbial community composition and maintains a balance between elemental uptake and release. The decomposition of soil organic matter is controlled by soil microbes, which affects the balance of C, N, and P in the ecosystem^10^. The microbial C:N:P ratio determines the direction of microbial activity and the release of organic nutrients^11^. Soil C, N, and P availability generally limit the metabolism of microorganisms^12–14^. Plant-microbe competition for nutrients increases when microbial nutrients are limited, posing a threat to plant colonization and growth^15,16^. Therefore, it is important to understand the relationship between the soil C:N:P ratio and the microbial C:N:P ratio to understand soil microbial nutrient limitations^17^.

Additionally, the enzyme stoichiometric ratio reflects the metabolic functions of microbial community and biological cycle of nutrients in the environment^18–20^. The enzyme stoichiometric ratio is calculated as an indicator of the C, N, and P requirements of soil microorganisms^21,22^. The interactions between various microorganisms and other factors, including temperature, moisture, N, P, and crop roots indirectly reflect the availability of soil resources^23,24^ and are an effective indicator for evaluating limitations in soil microbial resources^12–14^. The most commonly used methods to characterize limitations of soil microbial resources are the ratio of C, N, and P-related enzyme activities, enzyme stoichiometric vector analysis (vector length and vector angle), and threshold element ratios (TERC/N and TERC/P)^2,7,25^. Enzyme stoichiometry has been used to report patterns of soil resource limitation in cultivated land from various Chinese regions. For example, Cui et al.^2^ determined that the C:N:P ratio of microbial enzyme activities, the vector angle, and the threshold elemental C/N and C/P ratios of farmland in Jilin Province, China indicate that microbial metabolism is mainly N-limited under organic fertilizer treatments. Wang et al.^25 ^observed that a combined application of organic and chemical fertilizers reduces the soil carbon limitation in tobacco-planted soils in Yunnan based on a study of vector length and angle. Furthermore, some evidence suggests that there may be a dynamic equilibrium between the stoichiometric ratio of soil extracellular enzymes and the stoichiometric ratio of soil and microorganisms^26^. The stoichiometric ratio of enzymes remains relatively stable in areas with relatively restricted environments, and microorganisms maintain the balance between the acquisition and investment of various elements to cope with nutrient deficiencies and maintain soil nutrient equilibrium^27^. Yin et al.^4^ studied soil stoichiometry, microbial biomass, and soil enzymes in Northeast China, and reported that the enzyme N/P ratio was significantly negatively correlated with the soil N/P ratio, while the enzyme C/N ratio was significantly positively correlated with microbial biomass C/N. Therefore, an integrated analysis of the C, N, and P stoichiometric characteristics of soil resources, and microbial and enzymatic activities is necessary to study ecological chemometrics.

Applying fertilizer is key in maintaining and improving the fertility of agricultural soils. The nutrient content of farmland soil changes with fertilization. These changes alter the soil C, N, and P stoichiometry, and greatly affect the C, N, and P of soil enzyme activities and microbial biomass^13,28,29^. The imbalance between soil microbial demand and soil substrate supply affects C, N, and P nutrient cycling^9^. N is usually the key nutrient limiting the net primary productivity of agroecosystems^30^, and applying organic fertilizer can aggravate^5^ or diminish^6^ N limitation. For example, Shen et al.^5^ observed that replacing 20% or 50% of the chemical fertilizer combined with organic fertilizer aggravates the soil microbial N limitation in greenhouse soils for vegetable cultivation. In arid and semi-arid regions, organic fertilizer alone or in combination with N fertilizer diminishes N limitation^6^. Recent studies have shown that P limitation is common in agroecology^31,32^, and applying organic N to replace chemical fertilizer N helps relieve soil microbial C and P limitations^33^. Tobacco is an important commercial crop in Yunnan Province, and its yield and quality are affected by many factors, including climate, fertilization management, crop rotation pattern, soil properties, and soil microorganisms^34–36^. Previous studies on C, N, and P stoichiometry were used to reflect flue-cured tobacco soil fertility levels^37^. However, to our knowledge, only a few studies have elucidated the effects of different fertilization treatments on microbial resource limitations in tobacco-planting soils ^13,25^. In contrast, many studies have confirmed that using microbial fertilizers rather than chemical fertilizers promotes the absorption and transformation of soil-available nutrients while reducing environmental pollution and improving soil fertility^38^. Nevertheless, fertilizer-mediated changes in the microbial nutrient limitations of tobacco-planted soil are poorly understood. Therefore, it is important to further understand how microbial nutrient limitations respond to different fertilization strategies.

The ecological stoichiometric properties of soil C, N, and P in different ecosystems are significantly affected by the sampling period. Qi et al.^7^ indicated that soil and microbial-related properties and their C:N:P ratios are more influenced by sampling stage than by forest type. Jin et al.^39^ showed that the C:N:P ratio of paddy soil is significantly higher during the jointing period than during the maturation period. These studies demonstrated that it would be helpful to determine the stoichiometric characteristics of C, N, and P during different sampling periods to better reflect soil nutrient requirements and understand how the plant elements change during different growth periods.

In this study, we investigated C, N, and P stoichiometric and microbial nutrient limitations by measuring the C, N, and P contents of available soil resources, microbial biomass, and soil hydrolytic enzyme activities during the tobacco growing season, and studied the response of microbial resource limitation to applications of microbial fertilizer. We addressed the following two questions: (1) Does applying microbial fertilizer lead to changes in the stoichiometric ratio of soil and microbial biomass C, N, and P, compared to conventional fertilizer applications? (2) What factors affect the limitation of microbial resources in tobacco-planting soils? Based on the known relationship between ecological stoichiometry and microbial resource limitations, we hypothesized that (1) soil microbial biomass stoichiometry would be strictly homeostatic and would not change with soil C:N:P; (2) different fertilizer applications would lead to changes in microbial resource limitations; and (3) microbial resource limitations would vary among growth periods.

## Materials and methods

### Ethics statement

The authors affirm that all methods were performed following the relevant guidelines and regulations.

### Study area, experimental design, and soil sampling

The experimental site located at the new Ganlanpo flue-cured tobacco technology test base, Mile, Hani-Yi Autonomous Prefecture of Honghe, Yunnan, China (103°27′E; 24°23′N, elevation 1451 m). The average annual rainfall, temperature, and sunshine hours were 990.4 mm, 18.8 °C, and 2,131.4 h, respectively. The soil type was red soil and the previous crop was wheat. The major soil properties of the field before transplanting were: pH 6.09; soil organic carbon (SOC), soil total nitrogen (TN), soil total phosphorus (TP), and total potassium contents of 15.20, 1.34, 1.03, and 5.37 g/kg, respectively; and soil alkali-hydrolyzed nitrogen, available potassium, and available P contents of 121.87, 14.76, and 193.41 mg/kg, respectively. This experiment was randomly distributed within the field, with three replicates of four treatments, including conventional fertilization (CK), conventional fertilization + microbial fertilizer (T1), 75% conventional fertilization + microbial fertilizer (T2), and microbial fertilizer alone (T3). The conventional fertilization treatment was a humic acid organic-inorganic compound fertilizer of 50 g/plant (N + P_2_O_5_ + K_2_O ≥33%, 8–5–20) and the microbial fertilizer was 80 g/plant (CociCoLi, Wuhan Kenuo Biotechnology Co., Ltd., Wuhan, China). The number of effective viable bacteria was > 200 million/g, organic matter ≥ 60%, and humic acid ≥ 10%. The microbial fertilizer was used as the base dressing before transplanting and the compound fertilizer was applied at transplant. The test variety was the local main variety K326. Base and top-dressing fertilizer applications, picking, and backing were done in line with local management methods^36^. The row spacing of the tobacco plants was 1.2 × 0.55 m, 1000 plants/acre, and each treatment plot had 60 plants or about 40 m^2^ (excluding the protected lines).

Rhizosphere soil samples were collected according to the method of Wang et al.^35^ during the root extending period (6–8 leaves), the flourishing period (13–14 leaves), the maturation period (3–5 days before harvest), and the harvest period, and named R, F, M, H, respectively. The rhizosphere soils from three similar growing tobacco plants for each fertilization treatment were mixed, sieved to 2 mm after removing impurities, stored in a sealed bag, and transported back to the laboratory for preservation within 24 hours. Each sample was divided into two parts; one was naturally dried to determine basic soil physicochemical properties, and the other was stored at − 20 °C to determine the soil microbial properties.

### Soil physical, chemical, and microbial properties

Soil water content (SWC) was calculated by the amount of loss after drying for 48 h using the NY/T1121.3-2006 method. Soil pH was measured in water (1:2.5 w/v) with a pH meter (PHS-3C) according to NY/T1377-2007. The SOC, TN, and TP contents were measured according to HJ 695-2014, NY/T 53-1987, and NY/T 88-1988, respectively. The soil microbial biomass contents of C, N, and P (MBC, MBN, and MBP) were measured according to the chloroform-fumigation-extraction method^40^, and the conversion factor E values of microbial biomass C, N, and P were 0.38, 0.57, and 0.40, respectively^41–44^. We also calculated a range of soil and microbial ratios, such as SOC/TN (sC/N), SOC/TP (sC/P), TN/TP (sN/P), MBC/MBN (mC/N), MBC/MBP (mC/P), and MBN/MBP (mN/P) in this study.

We measured the activities of four common C, N, and P-related hydrolytic enzymes, including β-1,4-glucosidase (BG), β-1,4-*N*-acetyl-glucosaminidase (NAG), leucine aminopeptidase (LAP), and acid phosphatase (ACP). BG and NAG activities were determined according to a previously described method^45^. LAP and ACP activities were measured using a physiological assay kit (Suzhou Keming Biological Technology Co., Ltd., Suzhou, China) according to the manufacturer’s manual. As reported by many studies, BG (NAG + LAP) and ACP were used for C-acquire enzyme activities (C-acq), N-acquire enzyme activities (N-acq), and P-acquire enzyme activities (P-acq)^13^. In addition, we calculated the stoichiometric ratios of C, N, and P microbial enzyme activities, including BG to (NAG + LAP) (eC/N), BG to ACP (eC/P), and (NAG + LAP) to ACP (eN/P)^13^. We also calculated the specific enzyme activity per unit of microbial biomass, such as BG/MBC (C-acq/MBC), (NAG + LAP)/MBN (N-acq/MBN), and ACP/MBP (P-acq/MBP) to represent the microbial enzyme activity coefficient^25^. Finally, we calculated the vector angle and the ratio of C, N, and P enzyme activity to characterize the enzyme stoichiometry^46^, and we calculated microbial stoichiometric homeostasis^7,47,48^.

### Statistical analysis

We used permutation multivariate analysis of variance (PERMANOVA) to determine the effect and significance of sampling time and the fertilization treatments and their interactions on soil indicators using the “vegan” package in R^49^. We used the “shapiro.test” and “levene.test” packages to test the normality of the distribution and the homogeneity of variance, respectively. A logarithmic or reciprocal transformation was carried out for the indicators that did not conform to a normal distribution. Differences between groups were detected using the Kruskal–Wallis nonparametric test for the indicators that could not be transformed. One-way analysis of variance and Tukey's honestly significant difference (HSD) test were used to determine differences in soil basic physicochemical properties, soil, microbial, and related enzyme C, N, P stoichiometric ratios, and microbial resource limitation-related indicators between the fertilization treatments at the same sampling time^13^. The relationships between the microbial resource limitation (vector angles in this study) and soil physical properties, microbial biomass C, N, and P, and their stoichiometric ratios were analyzed by linear regression using the “ggpmisc” package in R^50^. A heatmap of the correlation coefficients in the “corrplot” package was used to assess the correlation between soil, microbial biomass, and enzymatic C:N:P. Principal component analysis (PCA) was conducted to determine the effects of sampling time and the fertilization treatments on soil microbial biomass and enzymatic C:N:P using the “prcomp” function in R^13^. Statistical analysis and graphing were completed using RStudio software package v.4.2.1.

## Results

### Effects of different sampling times and fertilization treatments on soil microbial biomass, enzymes, and the C:N:P stoichiometric ratios

SOC, TN and TP were not affected by the interaction between the sampling period and the fertilization treatment, or by either alone (Table 1). TN and TP were highest in the T2 treatment during the H period (Table 2). SWC and soil pH were significantly affected by sampling time and were lowest during the H period. Except for N-acq, all other microbial traits were affected by the sampling time (Table 1). MBC was highest during the F period, and MBN and MBP were highest during the H period (Table 2).

**Table 1:** Permutational multivariate analysis of variance (PERMANOVA) to assess the effects of fertilization treatment, sampling period, and their interactions on soil resources and C, N, and P stoichiometry; and enzymatic angle vectors.

	Treatment		Sampling periods		Treatment * sampling periods	
	F	*p*	F	*p*	F	*p*
SWC (%)	1.734	0.171	61.432	**0.001**	0.801	0.629
pH	4.904	**0.008**	16.043	**0.001**	2.106	0.060
SOC (g/kg)	1.201	0.316	1.745	0.196	0.830	0.608
TN (g/kg)	1.611	0.221	1.532	0.237	1.161	0.345
TP (g/kg)	1.446	0.265	1.558	0.229	0.535	0.883
C-acq (umol/d/g)	0.718	0.570	17.760	**0.001**	5.883	**0.001**
N-acq (umol/d/g)	0.511	0.665	2.586	0.070	1.226	0.301
P-acq (umol/d/g)	0.591	0.616	7.244	**0.003**	0.317	0.956
MBC (mg/kg)	0.740	0.531	8.818	**0.002**	2.007	0.064
MBN (mg/kg)	0.644	0.624	3.178	**0.036**	2.691	**0.023**
MBP (mg/kg)	0.404	0.760	4.424	**0.009**	0.567	0.829
C-acq/MBC	0.471	0.695	8.158	**0.001**	2.888	**0.009**
N-acq/MBN	1.456	0.268	11.798	**0.001**	2.901	**0.014**
P-acq/MBP	0.392	0.765	4.560	**0.012**	0.972	0.476
sC/N	0.426	0.758	1.316	0.281	0.375	0.944
sC/P	0.194	0.906	2.556	0.072	0.345	0.952
sN/P	0.068	0.965	0.702	0.549	0.338	0.959
mC/N	1.400	0.253	1.388	0.247	0.836	0.591
mC/P	1.374	0.289	7.301	**0.001**	0.561	0.865
mN/P	0.956	0.441	4.104	**0.027**	0.934	0.508
eC/N	0.363	0.765	7.488	**0.001**	3.535	**0.012**
eN/P	0.325	0.815	6.869	**0.001**	0.540	0.842
eC/P	0.497	0.690	11.930	**0.001**	5.074	**0.001**
Vector angle	0.403	0.750	6.477	**0.001**	0.570	0.817

SWC, soil water content; pH, soil pH; SOC, soil organic carbon; TN, soil total nitrogen; TP, soil total phosphorus; C-acq, BG; N-acq, NAG + LAP; P-acq, ACP; MBC, microbial biomass C; MBN, microbial biomass N; NBP, microbial biomass P; C-acq/MBC, C related enzyme activity to microbial biomass C; N-acq/MBN, N related enzyme activity to microbial biomass N; P-acq/MBP, P related enzyme activity to microbial biomass P; sC/N, SOC/TN; sC/P, SOC/TP; sN/P, TN/TP; mC/N, MBC/MBN; mC/P, MBC/MBP; mN/P, MBN/MBP. eC/N, BG/(NAG + LAP); eC/P, BG/ACP; eN/P, (NAG + LAP)/ACP. The vector angle represents soil N and P limits for microorganisms.

Significant values are in bold.

**Table 2:** Soil physicochemical properties and biological indicators across treatments during the flue-cured tobacco growing period.

Treatment	SWC (%)	pH	SOC (g/kg)	TN (g/kg)	TP (g/kg)	C-acq (umol/d/g)	N-acq (umol/d/g)	P-acq (umol/d/g)	MBC (mg/kg)	MBN (mg/kg)	MBP (mg/kg)
R-CK	0.25 ± 0.02a	6.77 ± 0.07ab	15.30 ± 0.76a	1.27 ± 0.01a	1.08 ± 0.03a	7.72 ± 0.71a	15.64 ± 1.94a	18.88 ± 1.26a	49.97 ± 1.70a	3.11 ± 0.04a	1.40 ± 0.29a
R-T1	0.23 ± 0.01a	6.90 ± 0.06a	17.83 ± 1.47a	1.47 ± 0.07a	1.21 ± 0.12a	6.09 ± 0.86a	17.95 ± 1.12a	18.00 ± 1.65a	72.68 ± 5.64a	5.48 ± 1.89a	2.90 ± 1.08a
R-T2	0.27 ± 0.02a	6.40 ± 0.15b	15.83 ± 1.03a	1.33 ± 0.12a	1.10 ± 0.03a	5.75 ± 0.68a	14.86 ± 1.82a	18.15 ± 1.68a	59.07 ± 5.43a	4.40 ± 1.26a	1.15 ± 0.30a
R-T3	0.26 ± 0.02a	7.00 ± 0.00a	15.20 ± 0.46a	1.32 ± 0.05a	1.05 ± 0.06a	5.58 ± 0.08a	17.13 ± 0.92a	18.11 ± 1.85a	53.35 ± 9.69a	2.53 ± 0.08a	0.96 ± 0.31a
F-CK	0.22 ± 0.02a	6.67 ± 0.03a	16.90 ± 1.21a	1.48 ± 0.10a	1.09 ± 0.01a	7.08 ± 1.39a	15.23 ± 1.72a	21.89 ± 1.62a	68.20 ± 10.57a	7.02 ± 1.65a	1.60 ± 0.39a
F-T1	0.22 ± 0.02a	6.83 ± 0.27a	16.43 ± 0.98a	1.33 ± 0.04a	1.09 ± 0.04a	5.13 ± 0.22a	14.73 ± 1.84a	21.60 ± 1.88a	53.92 ± 1.50a	1.53 ± 0.49a	3.52 ± 0.70a
F-T2	0.25 ± 0.01a	6.73 ± 0.20a	15.87 ± 0.66a	1.37 ± 0.04a	1.14 ± 0.02a	5.81 ± 0.26a	17.45 ± 0.29a	21.62 ± 1.00a	59.99 ± 6.71a	3.84 ± 1.77a	2.20 ± 0.98a
F-T3	0.20 ± 0.02a	6.63 ± 0.12a	15.73 ± 0.92a	1.26 ± 0.08a	1.07 ± 0.05a	6.20 ± 1.30a	13.93 ± 1.43a	22.37 ± 0.07a	54.13 ± 6.34a	4.38 ± 1.90a	1.94 ± 1.00a
M-CK	0.15 ± 0.01a	6.73 ± 0.15a	15.33 ± 0.81a	1.26 ± 0.02a	1.08 ± 0.04a	3.83 ± 0.40b	12.94 ± 1.07a	21.80 ± 1.21a	32.01 ± 8.22a	2.04 ± 0.13b	2.25 ± 0.95a
M-T1	0.13 ± 0.01a	6.90 ± 0.06a	14.87 ± 0.20a	1.26 ± 0.05a	1.09 ± 0.04a	3.67 ± 0.33b	13.75 ± 0.47a	21.12 ± 1.24a	38.69 ± 2.96a	2.19 ± 0.60ab	3.62 ± 1.82a
M-T2	0.14 ± 0.02a	6.47 ± 0.03a	15.27 ± 0.87a	1.33 ± 0.00a	1.09 ± 0.03a	3.66 ± 0.17b	14.63 ± 0.88a	21.80 ± 1.72a	35.16 ± 3.62a	2.42 ± 0.88ab	2.58 ± 0.81a
M-T3	0.13 ± 0.01a	6.93 ± 0.15a	14.40 ± 0.81a	1.25 ± 0.06a	1.08 ± 0.06a	8.42 ± 1.40a	13.65 ± 0.63a	20.48 ± 1.79a	40.69 ± 10.83a	5.26 ± 0.89a	3.44 ± 1.27a
H-CK	0.14 ± 0.00a	6.40 ± 0.00a	14.83 ± 0.47a	1.27 ± 0.03a	1.09 ± 0.04a	7.81 ± 0.14b	14.50 ± 0.23a	23.20 ± 1.36a	44.12 ± 9.17a	3.26 ± 0.91b	5.28 ± 0.59a
H-T1	0.13 ± 0.01a	6.03 ± 0.07b	15.50 ± 1.05a	1.36 ± 0.05a	1.21 ± 0.10a	9.60 ± 0.42a	12.27 ± 0.54a	20.26 ± 0.55a	45.91 ± 11.55a	6.00 ± 0.78ab	3.47 ± 0.06a
H-T2	0.13 ± 0.01a	6.17 ± 0.03ab	16.70 ± 0.65a	1.46 ± 0.09a	1.22 ± 0.02a	9.65 ± 0.21a	15.45 ± 2.58a	23.57 ± 1.15a	68.47 ± 5.45a	6.57 ± 0.66ab	5.41 ± 2.20a
H-T3	0.11 ± 0.01a	6.47 ± 0.13a	14.97 ± 0.57a	1.33 ± 0.03a	1.14 ± 0.07a	6.77 ± 0.14b	16.57 ± 1.892a	22.86 ± 0.37a	70.95 ± 7.21a	9.01 ± 1.44a	3.62 ± 0.33a

SWC, soil water content; pH, soil pH; SOC, soil organic carbon; TN, soil total nitrogen; TP, soil total phosphorus; C-acq, BG; N-acq, NAG + LAP; P-acq, ACP; MBC, microbial biomass C; MBN, microbial biomass N; NBP, microbial biomass P. R, F, M, and H indicate the root extending, flourishing, maturing and harvesting sampling periods, respectively. CK, conventional fertilization; T1, conventional fertilization + microbial fertilizer; T2, 75% conventional fertilization + microbial fertilizer; T3, microbial fertilizer. Values are mean ± standard error (n = 3). Lowercase letters indicate significant differences among the fertilizer treatments for each growing period (Tukey’s HSD test, *p* < 0.05).

**Figure 1:**
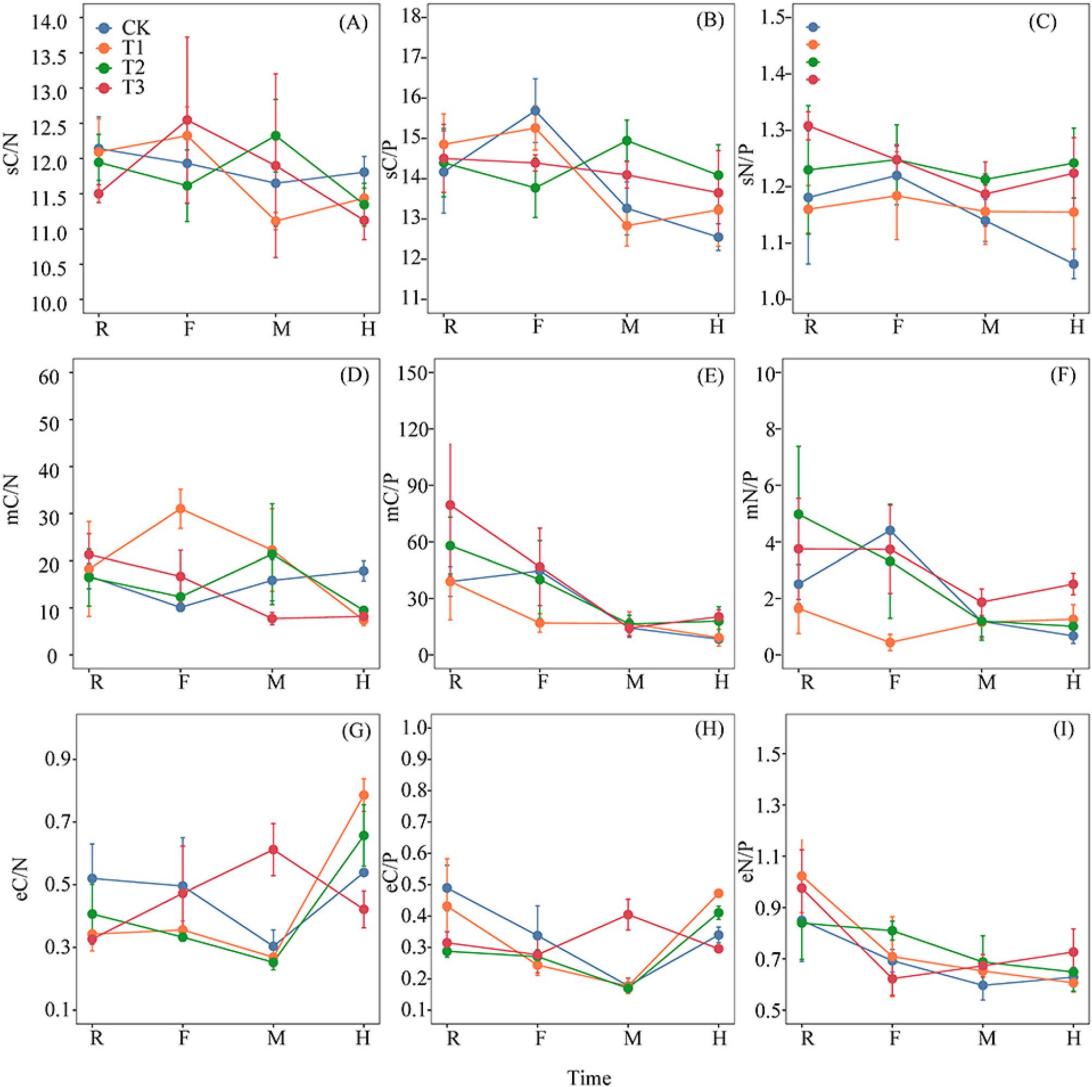
C:N:P stoichiometry of soil, microbial biomass and related enzyme activities during the sampling periods under different fertilization treatments. sC/N, SOC/TN; sC/P, SOC/TP; sN/P, TN/TP; mC/N, MBC/MBN; mC/P, MBC/MBP; mN/P, MBN/MBP. eC/N, BG/(NAG + LAP); eC/P, BG/ACP; eN/P, (NAG + LAP)/ACP. R, F, M, and H indicate the root extending, flourishing, maturation, and harvesting sampling periods, respectively. CK, conventional fertilization; T1, conventional fertilization + microbial fertilizer; T2, 75% conventional fertilization + microbial fertilizer; T3, microbial fertilizer. Values are mean ± standard error (n = 3).

Only eC/N and eC/P were significantly affected by the interaction between fertilization treatment and sampling time (*p* < 0.05). mN/P, mC/P, eC/N, eC/P, and eN/P were strongly affected by sampling time (*p* < 0.05) (Table 1). sC/P and sN/P were highest in the T2 treatment during the M and H periods (Fig. 1A–C). mC/N was highest in the CK treatment during the H period (Fig. 1D). mC/P and mN/P were highest in the T3 treatment (Fig. 1E–F). eC/N and eC/P were higher in the T3 treatment than in the other treatments during the M period, but the contents were highest in the T1 treatment during the H period (*p* < 0.05) (Fig. 1G,H). No significant differences in eN/P were observed among the four treatments during any of the growth periods (*p* > 0.05) (Fig. 1I). Sampling time and fertilization treatments had no significant effect on soil microbial biomass or soil resources (*p* > 0.05), indicating soil homeostasis among the different fertilization treatments during the same period (Table 3).

**Table 3:** Homeostatic coefficients of soil microbial biomass and their stoichiometries.

Sampling time	Variable (x)	Variable (y)	1/H	*R*^2^	*p*	Grade
R	SOC	MBC	0.408	0.079	0.375	Strictly homeostatic
	TN	MBN	0.926	0.020	0.660	Strictly homeostatic
	TP	MBP	−20.000	0.240	0.106	Strictly homeostatic
	sC/N	mC/N	0.078	0.268	0.268	Strictly homeostatic
	sC/P	mC/P	0.091	0.228	0.228	Strictly homeostatic
	sN/P	mN/P	0.671	0.238	0.238	Strictly homeostatic
F	SOC	MBC	0.341	0.036	0.553	Strictly homeostatic
	TN	MBN	2.757	0.037	0.548	Strictly homeostatic
	TP	MBP	2.328	0.030	0.593	Strictly homeostatic
	sC/N	mC/N	−0.175	0.142	0.227	Strictly homeostatic
	sC/P	mC/P	0.092	0.105	0.303	Strictly homeostatic
	sN/P	mN/P	2.545	0.000	0.955	Strictly homeostatic
M	SOC	MBC	0.599	0.032	0.577	Strictly homeostatic
	TN	MBN	1.210	0.002	0.903	Strictly homeostatic
	TP	MBP	2.545	0.312	0.059	Strictly homeostatic
	sC/N	mC/N	−0.397	0.049	0.489	Strictly homeostatic
	sC/P	mC/P	5.977	0.017	0.687	Strictly homeostatic
	sN/P	mN/P	−1.530	0.064	0.428	Strictly homeostatic
H	SOC	MBC	0.215	0.003	0.871	Strictly homeostatic
	TN	MBN	0.613	0.003	0.877	Strictly homeostatic
	TP	MBP	0.704	0.000	0.999	Strictly homeostatic
	sC/N	mC/N	0.180	0.006	0.814	Strictly homeostatic
	sC/P	mC/P	0.169	0.052	0.477	Strictly homeostatic
	sN/P	mN/P	1.565	0.145	0.223	Strictly homeostatic

1/H is the slope of the regression line between ln (y) and ln (x), where x is the soil resource stoichiometric ratio (e.g., sC/N), and y is the microbial biomass carbon, nitrogen, and phosphorus stoichiometric ratio (e.g. mC/N). The regression relationship was not significant (*p* > 0.05) in this study, so microbial stoichiometry was “strictly homeostatic”. R, F, M, and H indicate the root extension, flourishing, maturation, and harvesting sampling periods, respectively. SOC, soil organic carbon; TN, soil total nitrogen; TP, soil total phosphorus; MBC, microbial biomass C; MBN, microbial biomass N; MBP, microbial biomass P; sC/N, SOC/TN; sC/P, SOC/TP; sN/P, TN/TP; mC/N, MBC/MBN; mC/P, MBC/MBP; mN/P, MBN/MBP.

### Soil C, N, and P cycle-related enzyme activities and microbial resource limitations

**Figure 2:**
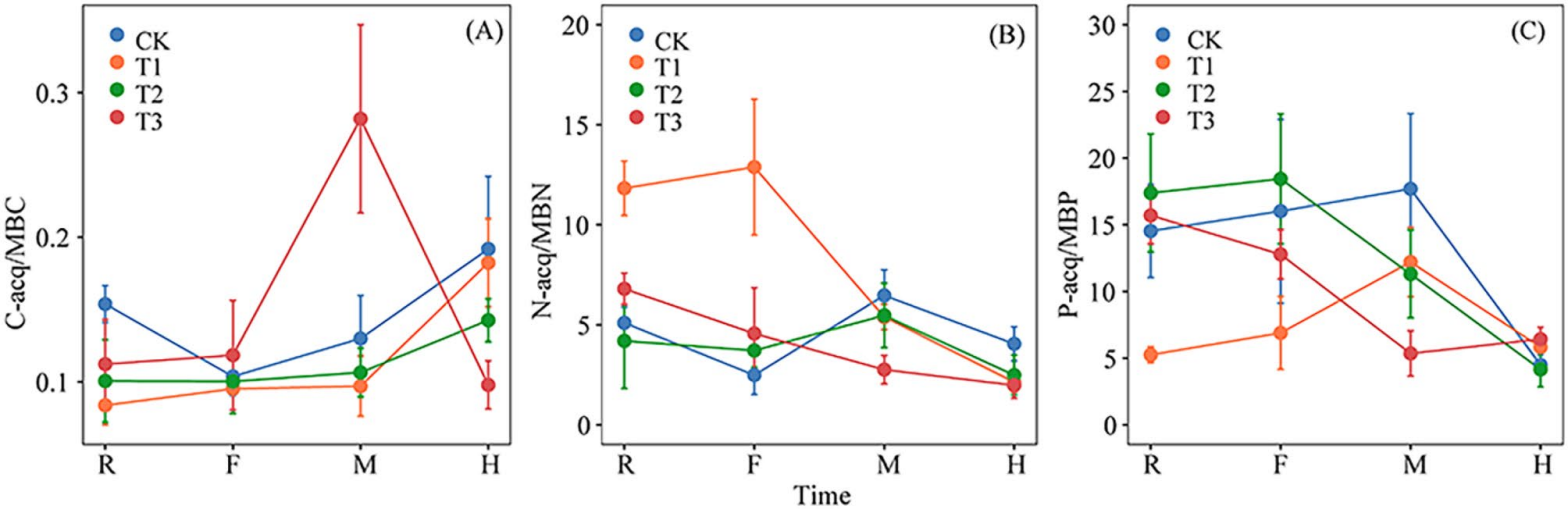
The ratio of soil C, N, and P related enzyme activities to microbial biomass C, N, and P (specific enzyme activity per microbial biomass unit: microbial enzyme activity coefficient) during the different sampling periods under different fertilization treatments. R, F, M, and H indicate the root extending, flourishing, maturation, and harvesting sampling periods, respectively. CK, conventional fertilization; T1, conventional fertilization + microbial fertilizer; T2, 75% conventional fertilization + microbial fertilizer; T3, microbial fertilizer. Values are the mean ± standard error (n = 3).

C-acq/MBC, N-acq/MBN, and P-acq/MBP were significantly affected by sampling time. C-acq/MBC and N-acq/MBN were also affected by the interaction between sampling time and fertilization treatment (*p* < 0.05) (Table 1). The C-acq/MBC for flue-cured tobacco was significantly higher in the T3 treatment during the M period (*p* < 0.05) (Fig. 2A). The N-acq/MBN and P-acq/MBP ratios were lowest during the H period (Fig. 2B,C).

**Figure 3:**
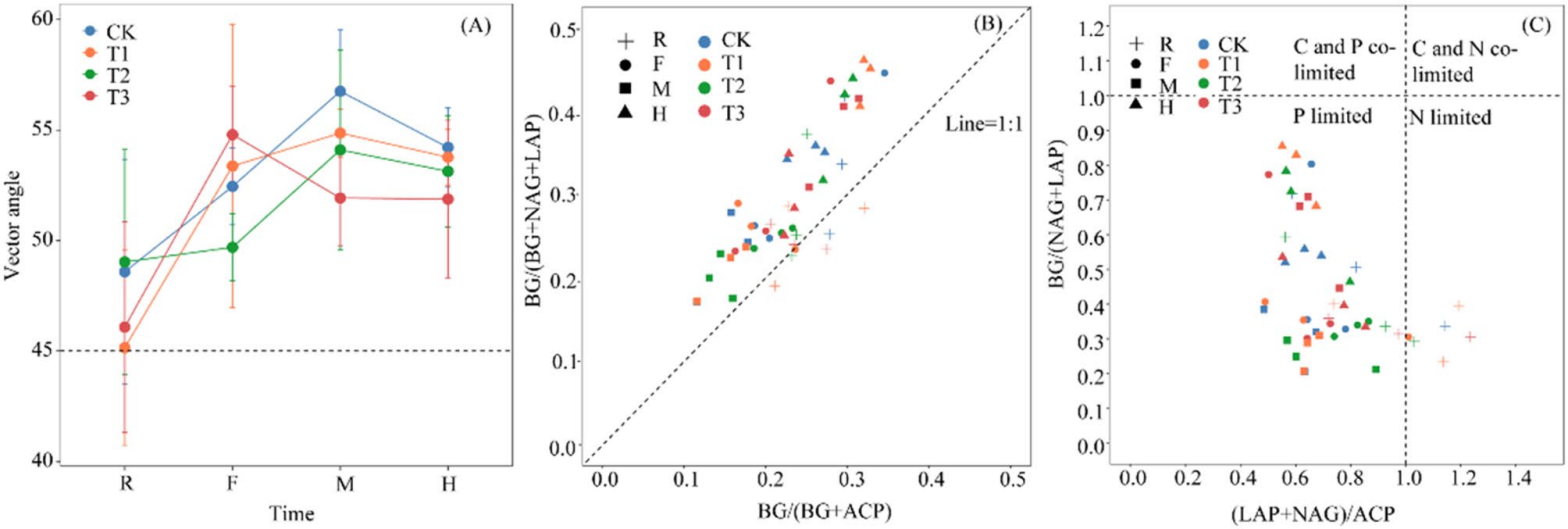
The vector angle (**A**) and soil extracellular enzyme stoichiometry (**B**, **C**). Vector angles < 45° indicate N limitations, whereas those > 45° indicate P limitations. R, F, M, and H indicate the root extending, flourishing, maturation, and harvesting sampling periods, respectively. CK, conventional fertilization; T1, conventional fertilization + microbial fertilizer; T2, 75% conventional fertilization + microbial fertilizer; T3, microbial fertilizer.

The vector angles of the four treatments at the different sampling times (*p* < 0.05) (Table 1) exceeded 45°, and the order during the M and H periods was T1 > T2 > CK > T3 (Fig. 3A). In contrast, almost all of the soil enzyme stoichiometry points were above the 1:1 line except for some samples from the R period (Fig. 3B), indicating that the samples were P limited except for N limitation during the R period. None of the soils was limited by C and N co-limitation or C and P co-limitation (Fig. 3C). Furthermore, the linear regression analysis shown in Table 4 indicated that the soil vector angle increased with SWC, pH, MBC, and mC/P (*p* < 0.05).

**Table 4:** Linear regression of soil physicochemical and microbial indicators with vector angles.

Variables (x)	Regression equation	*R*^2^	*p*
SWC	y = 59.2 − 39.8x	0.23	**0.001**
pH	y = 91.4 − 5.97x	0.14	**0.005**
SOC	y = 56.2 − 0.28x	0.01	0.556
TN	y = 54.7 − 2.14x	0.01	0.741
TP	y = 56.5 − 4.19x	0.01	0.585
MBC	y = 57.9 − 0.114x	0.13	**0.006**
MBN	y = 51.6 + 0.0553x	0.01	0.839
MBP	y = 50.5 + 0.459x	0.02	0.164
SCN	y = 57.5 − 0.479x	0.01	0.590
SCP	y = 52.1 − 0.0177x	0.01	0.974
SNP	y = 49.5 + 1.96x	0.01	0.772
mC/N	y = 53.4 − 0.1x	0.04	0.110
mC/P	y = 54.2 − 0.0778x	0.18	**0.001**
mN/P	y = 53.3 − 0.62x	0.05	0.064

SWC, soil water content; pH, soil pH; SOC, soil organic carbon; TN, soil total nitrogen; TP, soil total phosphorus; C-acq, BG; N-acq, NAG + LAP; P-acq, ACP; MBC, microbial biomass C; MBN, microbial biomass N; MBP, microbial biomass P. Significant values are in bold.

### Correlations between the soil, soil microbial biomass, and enzyme-related C, N, and P stoichiometric ratios

**Figure 4:**
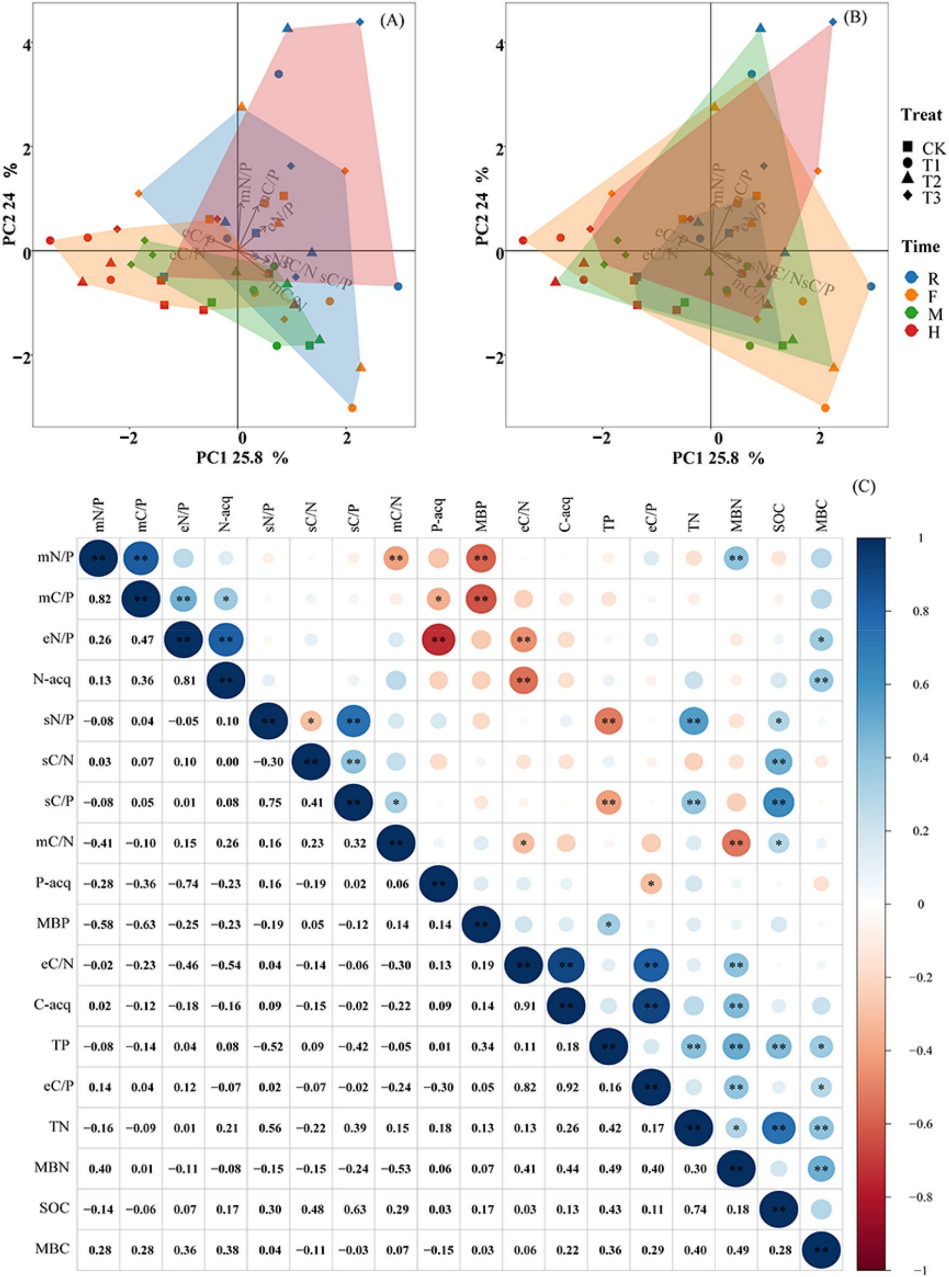
Principal component analysis (PCA) of soil resources, soil microbial biomass, and enzyme-related stoichiometric ratios of C, N and P (**A**, **B**) and correlation between the soil physicochemical and microbial indicators (**C**). R, F, M, and H indicate the root extending, flourishing, maturation, and harvesting sampling periods, respectively. CK, conventional fertilization; T1, conventional fertilization + microbial fertilizer; T2, 75% conventional fertilization + microbial fertilizer; T3, microbial fertilizer. Blue and red represent positive and negative correlations, respectively. The darker the color, the stronger the relationship. *significant at *p* < 0.05; **significant at *p* < 0.01; ***significant at *p* < 0.001.

The PCA results showed that axes 1 and 2 explained 25.8% and 24.0%, respectively, of the variation in soil resources, microbial biomass, and enzyme stoichiometry. The differences in the soil and microbial C, N, and P indices at the different sampling times were greater than the differences between fertilization treatments (Fig. 4A,B). The differences during the R and F periods were higher than those during the M and H periods (Fig. 4A). The difference in CK was lower than that in the other treatments with added microbial fertilization (Fig. 4B).

The correlation analysis further showed no significant relationship between mC/N and sC/N, mC/P and sC/P, or mN/P and sN/P. However, the soil eC/P and eC/N, mC/P and mN/P, and sC/P, and sC/N were positively correlated, as positive correlations were detected between MBC and SOC, MBN and TN, and MBP and TP (Fig. 4C).

## Discussion

The stoichiometric balance in soil resources is critical for maintaining microbial metabolism and a dynamic balance among the elements, which reflecting the ability of microorganisms to decompose soil organic matter and release P and indicating the supply of soil nutrients during plant growth^8,28,51^. Consistent with previous studies, soil SOC, TN, and TP contents in this study were significantly positively correlated (*p* < 0.05), and an interaction was detected between SOC, TN, and TP^28,52^. Tian et al.^28^ reported that the mean soil C/N, C/P, and N/P values in China were 11.9, 61, and 5.2, respectively. The average C/N value (11.45) in this study was similar to the above-average value, considering that carbon is a structural element, and its accumulation and consumption are relatively steady^53^. The variability of soil C/N in the different fertilization treatments among sampling times was not significant in this study. The average C/P and N/P values were 14.1 and 1.2, which were lower than the average soil values in China, possibly due to the low organic carbon content in red soil in this study^54^, or the lower pH and N availability^55^. However, adding microbial fertilizer improved the soil C/P and N/P values during the H period (Fig. 1B,C), possibly because *Bacillus subtilis* was contained in the microbial fertilizer, which improved soil N fixation capacity and SOC content^56^; *Bacillus mucilaginosus* decreases soil P content^57^. Interestingly, the reduced usage of compound fertilizer combined with microbial fertilizer (T2) in this study had a larger effect on increasing soil C/P and N/P (Fig. 1B,C).

Microbial resource limitations describe microbial growth and activity that is limited by nutrient availability and energy^58^. Ecological stoichiometry theory suggests that the C:N:P ratio of soil microbial biomass is more stable relative to the soil C, N, and P stoichiometry ratio and reflects the state of microbial C, N, and P demand^59^. Our results indicate no significant correlation between the microbial biomass stoichiometric ratio and the soil resources stoichiometric ratio (Fig. 4C). The strict homeostasis of soil microbial biomass between the fertilization treatments and different sampling times also confirmed the stability of microbial stoichiometry^7^ (Table 3), which supports our first hypothesis. Moreover, the global average values of mC/N, mC/P, and mN/P are 7.6, 42.4, and 5.6, respectively^52^. The mC/P and mN/P values were 30.52 and 2.37 in this study, which was lower than the global levels. This result indicates that soil microorganisms have a weak tendency to assimilate soil available P, and the ability to absorb P results from competition with plants^60^. However, the mC/N value (19.14) was higher than the global level, suggesting a relatively strong N fixation ability of the soil microorganisms in this study ^61^. The mC/N value was relatively stable in this study compared with a previous study^1^, and mC/P and mN/P varied more among the sampling periods (Fig. 1A), indicating greater stoichiometric plasticity in microbial P^1^. In contrast to a previous study, Qi et al.^7^ showed that soil mC/P and mN/P values were highest during the middle and late stages of forest vegetative growth (August). Our results show that the mC/P and mN/P values were highest during the R period, and lower during the M and H periods, which may be related to the different ecosystem and soil types^7^.

Previous studies have indicated that the ratio of global soil C, N, and P-related enzyme activities is 1:1:1^20^. A ratio that deviates from 1:1:1 suggests that soil microorganisms are affected by C, N, or P limitations^20^. The C:N:P ratio of the enzyme activities in this study was 1:1.45:1.64, indicating that soil microorganisms were more restricted by N and P than soil C. In addition, the enzyme stoichiometry points were mostly above the 1:1 line, and the vector angles in almost all treatments were greater than 45°, showing that P was limited, except for a few points where N was limited during the R period. Moreover, the soil microorganisms changed from N-limited to P-limited with the extension of the growing period^46^ (Fig. 3A). Notably, enzymatic stoichiometry is controversial for determining carbon resource constraints^25,31^. However, our study combined C, N, and P enzyme stoichiometric characteristics and the vector angle to determine microbial resource limitations, took place on tobacco planting soil that was limited by N and P, which can minimize this bias, and yielded convincing results. The soil N and P limitations may be due to the acidic soil in this study. Previous research has suggested that P limitations are mainly due to the strong binding of Fe^3^^+^ and Al^3+^or that water-soluble P is slowly converted to occluded P in acidic soil, resulting in reduced P utilization^62,63^. Secondly, P limitation increased first and then decreased as the growing period of flue-cured tobacco was extended. The T3 treatment had an earlier weakening trend, and weakened from the F to the M period, while the remaining treatments showed a weakening trend from the M to the H period. The changes in P limitation may have occurred because a large amount of P is needed to supply flue-cured tobacco primary productivity during the vigorous growing period, thereby increasing the P limitation of soil microorganisms^13^, and P limitation was alleviated by increasing the soil total P during the H period^64^ (Table 2). The results also show that the full application of microbial fertilizer (T3) had a more obvious effect on alleviating P limitation, which was conducive to the microbial nutrient balance by alleviating competition for nutrients between soil microbes and the soil. Herein, our results support the second and third hypotheses that different fertilizer applications lead to changes in microbial resource limitations, which varied during different growth periods.

Moreover, Yang et al.^12^ showed that microbial N and P limitations are affected by the soil nutrient stoichiometric ratio, soil water content, soil pH, soil bulk density, and SOC. At the same time, other studies have shown that temperature, soil moisture, soil pH, and SOC affect microbial P limitations^12,65^. In this study, SWC, soil pH, MBC, and mC/P had significant negative effects on the microbial N and P limitations (Table 4). Consistent with previous results, higher SWC accelerates the decomposition of SOC^12^, which enhances microbial activity and microbial biomass carbon content, and the soil nutrient limitation converts from an N limitation to a P limitation^66^. However, low soil pH and mC/P lead to reduced availability of P in soil, so microorganisms compete with plants for P, and biological fixation of P occurs, thereby aggravating the P limitation^62,65^. In this study, SWC, soil pH, and soil mC/P decreased as the growth period was extended, which may have resulted in the weakening of P limitation during the H period (Table 2).

## Conclusions

Soil resources, microbial biomass, enzyme activities, and stoichiometric ratios were generally more affected by sampling time than by fertilization treatment. The stoichiometric ratio of microbial C, N, and P was strictly homeostatic and was not affected by changes in the soil C, N, and P stoichiometric ratio. While the soil microbial metabolism in tobacco-planting soils during different growth periods was more susceptible to restricted P, SWC, soil pH, MBC, and mC/P were the key factors affecting the P limitation. We also found that adding microbial fertilizer reduced P limitation during the M and H periods. This study links soil physicochemical properties with soil microbial metabolic limitations, which will deepen our understanding of soil nutrient cycling mechanisms.

## Data Availability

All data generated and analyzed in this study are included in this published article.
